# Suicide and Self‐Harm in Intellectual Disability: A Systematic Review and Meta‐Analysis

**DOI:** 10.1111/jir.70052

**Published:** 2025-10-04

**Authors:** Sara Lindstedt, Christian Rück, Tatja Hirvikoski, Emma Hintze, Johan Lundin Kleberg, Leoni Grossmann, John Wallert, Johan Bjureberg, Oskar Flygare

**Affiliations:** ^1^ Centre for Psychiatry Research, Department of Clinical Neuroscience Karolinska Institutet, & Stockholm Health Care Services, Region Stockholm Stockholm Sweden; ^2^ Department of Women's and Children's Health, Pediatric Neuropsychiatry Unit Center for Neurodevelopmental Disorders at Karolinska Institutet, Karolinska Institutet Stockholm Sweden; ^3^ Habilitation and Health, Stockholm Health Care Services, Region Stockholm Stockholm Sweden; ^4^ Department of Psychology Stockholm University Stockholm Sweden

**Keywords:** intellectual disability, meta‐analysis, self‐harm, suicide, systematic review

## Abstract

**Background:**

Individuals with intellectual disabilities (ID) are disproportionately exposed to several risk factors for suicidality. However, no meta‐analysis has yet quantified the relative risk of suicide and self‐harm, including suicide attempts, within this population. The aim of this project was to bring together and synthesise the research on suicidality among individuals with ID.

**Methods:**

A systematic review and meta‐analysis was carried out. Medline, Embase, Web of Science and PsycInfo were searched from inception through 4 August 2025. Observational studies with a quantitative design, evaluating the relative risk of suicide or self‐harm, including suicide attempts, in individuals with and without ID, were included. Risk of bias was assessed using a shortened version of the Risk Of Bias In Non‐randomized Studies–of Exposure (ROBINS‐E) checklist. A random effects model was used to synthesise the results.

**Results:**

Eleven primary studies were included in the review (*n* = 241 438). The level of ID severity was only presented in two articles. Compared to the general population, the pooled relative risk for death by suicide was 0.54 (95% CI 0.33 to 0.89, *k* = 6, *I* ^2^ = 77%) and the relative risk for self‐harm was 3.16, (95% CI 2.3 to 4.35, *k* = 6, *I* ^2^ = 89%).

**Conclusion:**

The findings suggest that individuals with ID have an elevated risk of self‐harm but a lower risk of dying by suicide compared to the general population. However, these results should be interpreted with caution due to the limited number of primary studies and substantial between‐study heterogeneity. Further, separate analyses of mild versus moderate‐to‐profound ID are warranted.

## 1 Introduction

Intellectual disability (ID) is a neurodevelopmental disorder characterised by intellectual difficulties, along with corresponding impairments in adaptive behaviours. The intellectual difficulties involve impairments in cognitive abilities such as reasoning, problem solving, planning, abstract thinking, judgement, study skills and the ability to learn from experience (American Psychiatric Association 2013). The aetiology of ID is heterogenous and it can be due to genetic causes (e.g., Down syndrome) and environmental causes (e.g., obstetrical complications causing anoxia) (Lee et al. 2025). Individuals with ID are exposed to many risk factors for suicide and self‐harm (Chan and Bhandarkar 2025). For example, a recent meta‐analysis found that more than one third of adults with ID have a co‐occurring psychiatric diagnosis (Mazza et al. 2020). ID also often co‐occurs with other neurological impairments and medical disorders such as cerebral palsy, epilepsy and various genetic disorders, and individuals with ID have an elevated risk of obesity, diabetes and heart and respiratory disease, all of which are associated with an elevated suicide risk (Boat et al. 2015; Erlangsen et al. 2020; Fazel et al. 2013; WHO and UNICEF 2023). In addition, individuals with ID and co‐occurring psychiatric disorders are exposed to social determinants of suicide and self‐harm such as unemployment, low income, social isolation and interpersonal violence more often than typically developing individuals (McClelland et al. 2020; Qin et al. 2003; Sariaslan et al. 2020).

Given the challenge of ID and co‐occurring conditions, along with social factors, the small amount of literature on suicidality in this group stands out. A recent systematic review investigating the current state of knowledge on suicidality in individuals with ID concluded that there are significant gaps in the current understanding of suicidality in this population (Chan and Bhandarkar 2025). As ID often co‐occurs with other neurodevelopmental conditions such as autism, the impact of ID on suicidality is rarely studied in isolation (Blanchard et al. 2021; Dimian and Symons 2022; Hand et al. 2020; Soke et al. 2016). The available evidence suggests that individuals with ID have lower suicide mortality but a higher prevalence of self‐harm, including suicide attempts. However, the evidence remains limited, as previous systematic reviews have not conducted a meta‐analysis to quantify the difference, leaving the research findings fragmented and the overall understanding of the topic incomplete (Dodd et al. 2016; Huisman et al. 2018; Nagraj and Omar 2015).

Studying suicidality in the ID population presents two critical challenges in identifying and accurately classifying suicidal behaviour: suicidality in individuals with ID varies with cognitive functioning, and diagnostic overshadowing may lead to mistakenly attributing suicidal expression to the intellectual disability itself. Previous reviews have highlighted these challenges (Chan and Bhandarkar 2025; Dodd et al. 2016; Mason and Scior 2004). This complexity is also reflected in the choice of outcomes when studying suicidality in this population. Previous systematic reviews have focused on outcomes such as suicide death, suicide attempts and suicidal ideation (Chan and Bhandarkar 2025; Dodd et al. 2016), while excluding self‐harm due to its often non‐suicidal motivations in the ID population (Chan and Bhandarkar 2025). However, assessing the intention behind self‐harming behaviours is challenging due to communication difficulties and a tendency to for caregivers and clinicians to overlook intention, which may result in missing suicidal behaviours. For this reason, self‐harm was included in this systematic review and meta‐analysis.

The overall aim of this project is to bring together and synthesise the research on suicidality among individuals with ID and do a meta‐analysis of the relative risk of suicide and self‐harm among people with ID compared to the general population. The study population includes the entire population, comparing the rates of suicide deaths, suicide attempts, and self‐harm in individuals with and without ID. Suicidal ideation among people with ID has been explored in recent systematic reviews (Chan and Bhandarkar 2025; Dodd et al. 2016) and was not included in this review, since the aim was to evaluate epidemiological studies that included suicide and self‐harm as outcomes to complement existing reviews. A better understanding of suicide and self‐harm in people with ID can guide assessment practices, targeted prevention efforts and lead to improved interventions. Accurate estimates of the relative risk of suicide and self‐harm in this population are therefore crucial for clinical practice.

## 2 Methods

The review was carried out in line with the PRISMA guidelines and was pre‐registered on PROSPERO and Open Science Framework (OSF) before the search was conducted (OSF 2023; PROSPERO 2023).

### 2.1 Search Strategy

Eligibility criteria included observational studies with a quantitative design that define the exposure by a diagnosis of ID according to International Classification of Diseases (ICD) or Diagnostic and Statistical Manual of Mental Disorders (DSM) or other established methods (e.g., other types of assessments or diagnosis of Down syndrome), providing relative risk estimate of death by suicide or self‐harm, including suicide attempt, as an outcome, and where the data on exposure and outcome was linked together.

A literature search was performed in the following databases: Medline, Web of Science, Embase and PsycInfo. The initial search was conducted 2024‐05‐24 and was last updated 2025‐08‐05. The search strategy was developed in Medline (Ovid) in collaboration with librarians at the Karolinska Institutet University Library. For each search concept Medical Subject Headings (MeSH‐terms) and free text terms were identified. The search was then translated, in part using Polyglot Search Translator (Clark et al. 2020), into the other databases. No language restriction was applied. Databases were searched from inception. The strategies were peer reviewed by another librarian prior to execution. De‐duplication was done using the method described by Bramer et al. (2016). One final, extra step was added to compare DOIs. A snow‐ball search was applied to check references and citations of eligible studies from the database searches using Web of Science. The full details of the search strategy are outlined in the Supporting Information.

### 2.2 Selection Process

The selection process was carried out in two steps. In step one, the two reviewers applied eligibility criteria based on the articles' title and abstract. In step two, the selected articles from step one were read in full text. In both steps, the reviewers screened records for inclusion independently and were blinded to each other's decisions. Any discrepancies were discussed and resolved. In the first step, inter‐rater reliability was tested by Prevalence‐ And Bias‐Adjusted Kappa (PABAK) (Byrt et al. 1993). Strength of agreement was assessed as slight agreement (< 0.20), fair agreement (0.21–0.40), moderate (0.41–0.60), substantial agreement (0.61–0.81) or almost perfect (0.81–1.00) (Sim and Wright 2005). In the second step, potentially eligible articles were discussed between the reviewers to determine inclusion in the meta‐analysis. If two or more studies used the same data set, the study most relevant for the research question was included in the review. If data from studies were overlapping, the study with the largest data set was included. The software Zotero (Corporation for Digital Scholarship 2023) was used to delete duplicates. The software AS Review (van de Schoot et al. 2021) was used for the first screening step, and the software Covidence was used for the second screening step, and to record decisions (Innovation 2024).

### 2.3 Data Extraction and Coding

Relevant data from the included articles were extracted and coded in an excel sheet. Variables that were extracted included author, year of publication, country, number of participants with ID, sex, level of ID, type of suicidality outcome, type of outcome measure, relative risk estimate, adjustment of estimate, setting, method for determining suicidality outcome and method for determining ID.

For some studies, the data available was not sufficient to be included in the meta‐analysis. In these cases, reviewers derived estimates using the following techniques. In the cases where person years (not number of participants with ID) were reported (Strauss et al. 1998; Zilber et al. 1989), number of person years was divided with the follow‐up time. This involves the assumption that no one dies during the follow‐up time, resulting in an under‐estimation of the number of participants included. When only the number of the total population, but not the population with ID was reported, a prevalence of 1% was assumed (Maulik et al. 2011; Park et al. 2017). For one study (Cervantes et al. 2023), we calculated odds ratio by dividing the odds of an event in the group with ID by the odds of the event in the group without ID. Finally, for one study (Patja et al. 2001), where risk estimates were presented for males and females separately, the risk estimates were combined using the method from van Dooren et al. (2013). The standard error for the included studies was calculated from the confidence interval to prepare the data for the synthesisation (Higgins et al. 2003).

### 2.4 Risk of Bias Assessment

Risk of bias was assessed using a shortened version of the Risk Of Bias In Non‐randomized Studies–of Exposure (ROBINS‐E) checklist translated into Swedish by the Swedish Agency for Health Technology Assessment and Assessment of Social Services (SBU). The checklist assesses bias from (1) confounding factors, (2) exposure, (3) drop‐out rate, (4) outcome measure, (5) selective reporting and (6) conflicts of interests. The domains addressing selection bias and bias due to post‐exposure interventions were excluded. Studies received an overall level of bias: low, high or unacceptably high (ROBINS‐E Development Group 2023).

### 2.5 Statistical Analyses

The outcomes of interest were suicide death and self‐harm (including suicide attempts). The relative risk for suicide death and self‐harm among people with ID compared to the general population was synthesised in a random effects meta‐analysis. The primary studies presented different measures of risk, such as risk ratio, hazard ratio (HR) and standardised mortality ratio. All measures were considered equivalent measures of risk and are hereafter referred to as ‘relative risk’, like previous meta‐analyses in the field (Chen et al. 2024). Pooled relative risk was estimated with 95% confidence intervals and *p* value. The inverse variance method was used to calculate study weight. The pooled estimate was presented in a forest plot alongside results from the individual studies and study weight. When substantial heterogeneity was present (i.e., Higgins *I* ^2^ ≥ 50%), outliers (e.g., studies where the point estimate and 95% confidence intervals did not overlap with the overall effect) were removed (Mathias Harrer et al. 2021). Between‐studies variance (*τ* ^2^) was estimated with a restricted maximum‐likelihood estimator (Veroniki et al. 2016), and between‐study heterogeneity was estimated with Higgings *I* ^2^ with values 25%, 50% and 75% indicative of low, moderate and high heterogeneity, respectively (Higgins et al. 2003). Analyses were performed separately for risk of suicide and suicide attempts.

Publication bias was examined visually by inspecting a funnel plot and quantitatively by calculating Egger's regression test of asymmetry (Egger et al. 1997). Planned sensitivity analyses included an analysis only including studies with a low risk of bias, a leave‐one‐out analysis, and an analysis including the less adjusted models available. A trim and fill procedure to compensate for risk of publication bias was also planned (Duval and Tweedie 2000). The following variables were planned to be investigated as moderators: publication year, country of the sample, proportion of women, age of the ID sample, level of ID, type of relative risk measure, level of adjustments, clinical setting or not. Statistical analyses were performed using the *meta* and *dmetar* packages (Balduzzi et al. 2019; Harrer et al. 2019) in R, version 4.3.2 (R Core Team 2023). Scripts and data are shared on the OSF (masked during peer review).

## 3 Results

### 3.1 Study Selection

**FIGURE 1:**
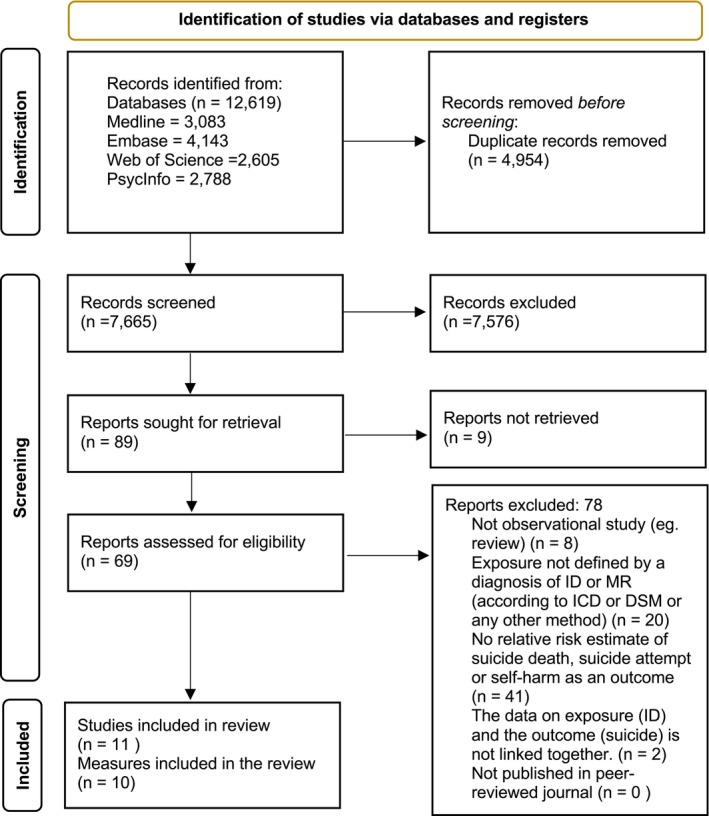
PRISMA flowchart of included studies.

The literature search yielded 12 619 potentially eligible articles, including duplicates. Once 4954 duplicates were removed, 7665 articles remained for title and abstract screening using the criteria in Table 1. The PABAK statistics at screening indicated almost perfect agreement (*k* = 0.95, 95% CI = 0.94–0.96). After screening, 89 articles were sought for retrieval. Nine articles were not accessible and therefore not included. Seventy‐eight articles were removed for the following reasons: eight because the data was not observational (e.g., a literature review), 20 because the exposure was not defined by a diagnosis of ID and 41 because they did not provide case–control data on the relative risk for suicide death, self‐harm (including suicide attempt) expressed as a relative risk (see Figure 1 for a PRISMA flowchart of the study selection).

**TABLE 1:** Characteristics of included studies.

Study	Country*	No of participants with ID	Gender ID**	Level ID	Type of suicidality outcome	Estimate (95% CI)	Adjustment description	Risk of bias	Setting	Method for determining suicidality outcome	Method for determining ID
Cervantes et al. (2023)	United States of America	90 372	n/a	n/a	Self‐harm (OR)	3.2 (3.05; 3.36)	No adjustment	Low	Clinical setting	ICD‐9 codes for intentional self‐harm (E950‐E959)	ICD‐9 codes of ID (317–319)
Erlangsen et al. (2020)	Denmark	35 266	n/a	n/a	Suicide death (IRR)	1.6 (1.3; 1.9) 0.6 (0.5–0.8)	Period, sex and age group. Adjusted model***	Low	Not clinical setting	Cause of death register	ICD‐8 codes of ID (311–315) and ICD‐9 codes of ID (F70–F79)
Flygare Wallén et al. (2023)	Sweden	11 969	43,75	n/a	Self‐harm (OR)	3.20 (2.66; 3.84)	Age	Low	Clinical setting	ICD 10 codes for intentional self‐harm (X60–X84) and undetermined intent of self‐harm (Y10–Y34)	ICD‐10 codes of ID (F70–F79) and congenital disorders associated with ID⊛
Jonsson et al. (2014)	Sweden	5152	46,18	n/a	Suicide attempt (HR)	2.03 (1.61; 2.55) 1.74 (1.38; 2.19) 1.72 (1.37; 2.17)	No adjustment Adjusted model† Extra adjusted model‡	Low	Not clinical setting	ICD‐10 codes for intentional self‐harm (X60‐X84)	ICD‐10 codes of ID (F70–F79)
Park et al. (2017)	Republic of Korea	10 253 ※	n/a	n/a	Suicide death (HR)	1.12 (0.6; 2.09) 1.28 (0.69; 2.39)	No adjustment Adjusted model§	Low	Not clinical setting	Cause of death register	Certificate of disability
Patja et al. (2001)	Finland	2369	50,02	n/a	Suicide death (SMR)	0.46 (0.23; 0.94) #	Age and gender	Low	Not clinical setting	Cause of death register	Psychological tests
Pouls et al. 2022	Netherlands	11 887	38,27	100%	Suicide attempt (OR)	3.8 (3.14; 4.6]	Age, sex and time in database	High	Clinical setting	ICPC‐P codes for suicide and suicide attempt (P77)	ICPC code of ID (P85) and registered mild ID diagnoses in database
Singhal et al. (2014)	United Kingdom	23 995	46	n/a	Self‐harm (RR) Suicide death (RR)	0.1 (0.1; 0.2) 0.4 (0.1; 1.3)	Adjusted model¶	High	Clinical setting	ICD‐9 (E950–E959) and ICD‐10 codes (X60–X64, X66–X84) for intentional self‐harm Hospital records and death registrations	ICD‐codes of Down syndrome (Q90)
Strauss et al. (1998)	United States of America	48 914	44	37% mild, 27% moderate, 36% severe/profound/other	Suicide death (SMR)	0.31 (0.21–0.43)	Age and gender	Low	Clinical setting	Cause of death register	Developmental disabilities database
Lundin et al. (2011)	Sweden	612	0	n/a	Suicide death (HR) Suicide attempt (HR)	3.5 (2.2; 5.5) 4.8 (3.5–6.5)	Age and gender	High	Not clinical setting	Cause of death register ICD‐8, 9 (E950‐E959) and 10 codes (X60‐X84) for intentional self‐harm	ICD‐8 codes of ID (310–315)
Zilber et al. (1989)	Israel	649	n/a	n/a	Suicide death (SMR)	3.27 (−3.14; 9.68)	Sex, age and ethnic origin	Low	Clinical setting	Cause of death register	ICD‐9 codes of ID (not specified)

* Country of the sample;

** = proportion of women;

*** = period, sex and age group, living status, region, socioeconomic status, physical comorbidity, psychiatric hospitalization prior to diagnosis of any neurological disorders and deliberate self‐harm prior to diagnosis of any neurological disorders;

= sex, country of birth, parental educational level and parental suicidal behaviour;

= history of suicide attempt (in addition to the adjusted model);

= age, sex, area of residence, household income and type of health care coverage;

= adjusted for age in 5‐year bands, time period in single calendar years, region of residence and neighbourhood socioeconomic disadvantage;

= calculated by combining men and women's SMR;

= calculated from total study population multiplied by presumed prevalence of ID;

= F89, Q91, Q92.1; Q92.2; Q92.3; Q92.6; Q93.4; Q93.5; Q99.2 (males only); Q87.1C; Q87.1F; Q87.8; Q87.2D; F84.2; F84.4.

Nine primary studies met all inclusion criteria, and two additional articles were added from the backward and forward reference search. Eleven primary studies were included in the review. However, the study by Zilber et al. (1989) had a small sample of individuals with ID and there was only one observed death, making it impossible to calculate a standard error. As a result, 10 studies were therefore included in the meta‐analysis, six measures for each outcome. Two studies appeared to meet the inclusion criteria but were excluded: Calver et al. (2021) was excluded because the exposure was defined as a combination of ID and autism, while Hosking et al. (2016) was excluded because suicide deaths were not reported separately from deaths by external causes.

### 3.2 Study Characteristics

The eleven studies included in the systematic review had an estimated total of 241 438 participants with ID. Two studies had a predominantly female sample, four had a predominantly male sample and one study consisted of only men (data from conscription registers). The rest of the studies did not present sex proportion for the ID group. The geographical origin of the studies spread over eight countries worldwide: Denmark, Sweden, Republic of Korea, Finland, United States of America, United Kingdom, Netherlands and Israel. The level of ID severity was only presented in two articles (Cervantes et al. 2023; Strauss et al. 1998), and differences in outcomes across ID severity levels were evaluated in just one study (Cervantes et al. 2023). One study (Pouls et al. 2022) only included individuals with mild ID. Five articles used a non‐clinical sample and six used a clinical sample (the setting was classified as clinical if the study took place in a clinical environment, such as a hospital). To determine a diagnosis of ID, seven studies used ICD codes from registers, one used International Classification of Primary Care (ICPC) codes and registered ID in a database, one used clinical testing and two studies included people based on certificates of disability or their existence in a disability database. Of the studies that used ICD codes, five used ICD codes of ID; one used codes for ID combined with codes for congenital disorders associated with ID, and one used only the code for Down syndrome.

Five studies reported only suicide death as the outcome measure, two only reported self‐harm, two reported suicide attempts, one study reported both suicide deaths and self‐harm and one study reported both suicide deaths and suicide attempts. The seven studies reporting suicide death as the outcome measure used cause of death registers to assess suicide. The three studies reporting self‐harm as the outcome measure used ICD codes, although the codes differed slightly: One of the three studies included both intentional self‐harm (X60–X84) and self‐harm with undetermined intent (Y10–Y34), and the others included only intentional self‐harm and one of them excluded Intentional self‐poisoning by and exposure to alcohol (X65). Of the three studies reporting suicide attempt as the outcome measure, two used ICD codes, and one used ICPC codes (P77), also including some cases of death by suicide. Regarding adjustment of estimate, one study did not adjust for any variables. One study only adjusted for age, three studies adjusted for age and sex and two studies additionally adjusted for time. Four studies adjusted for additional variables such as country of birth, ethnic origin, socioeconomic adjustments (region of residence and area of residence, socioeconomic status, living status, parental educational level, household income, type of health care coverage and neighbourhood socioeconomic disadvantage) and medical and psychiatric history (physical comorbidity, parental suicidal behaviour, psychiatric hospitalisation prior to diagnosis of any neurological disorders, deliberate self‐harm prior to diagnosis of any neurological disorders and history of suicide attempt). Three studies reported multiple levels of adjustments. Eight studies had an overall low risk of bias, and three studies had an overall high risk of bias. See Table 1 for a full list of study characteristics.

### 3.3 Relative Risk for Suicide in People With Intellectual Disability

**FIGURE 2:**
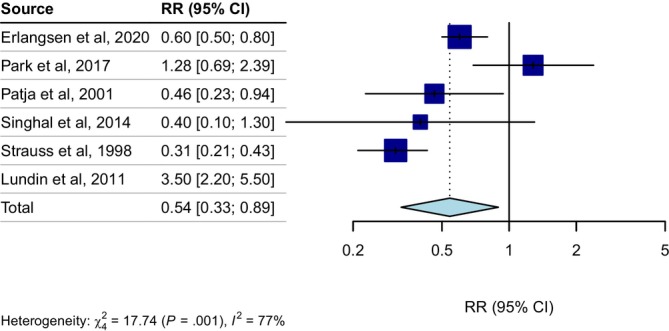
Relative risk of suicide for individuals with intellectual disabilities compared to the general population. Lundin et al. (2011) was excluded from the pooled estimate as it was identified as an outlier.

The random effects meta‐analysis of the six included studies reporting suicide death as the outcome measure did not show a difference in risk of suicide death among individuals with ID; Relative risk = 0.75, (95% CI 0.35 to 1.6, *n* = 121 409, *k* = 6, *I* ^2^ = 93%, *τ* ^2^ = 0.78, *p* = 0.462). The study by Lundin et al. (2011) was identified as an outlier and removed from further analysis, leaving five studies for the final meta‐analysis (Erlangsen et al. 2020; Park et al. 2017; Patja et al. 2001; Singhal et al. 2014; Strauss et al. 1998). Excluding the outlier, there was a lower relative risk of suicide death among individuals with ID compared to the general population, (RR = 0.54 [95% CI 0.33 to 0.89], *n* = 121 409, *k* = 5, *I* ^2^ = 77%, *τ* ^2^ = 0.23, *p* = 0.017), see Figure 2.

### 3.4 Relative Risk for Self‐Harm in People With Intellectual Disability

**FIGURE 3:**
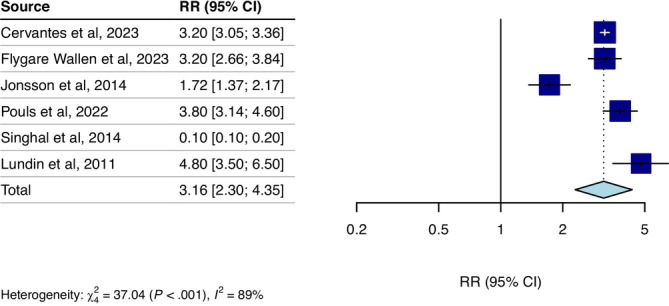
Relative risk of self‐harm for individuals with intellectual disabilities compared to the general population. Singhal et al. (2014) was excluded from the pooled estimate as it was identified as an outlier.

The random effects model on the six included studies reporting self‐harm as the outcome measure did not show a difference in risk of self‐harm among individuals with ID; relative risk = 1.79, (95% CI 0.56 to 5.68, *n* = 141 332, *k* = 6, *I* ^2^ = 99%, *τ* ^2^ = 2.07, *p* = 0.322). The study by Singhal et al. (Singhal et al. 2014) was identified as an outlier, thus leaving five measures for the final meta‐analysis (Cervantes et al. 2023; Flygare Wallén et al. 2023; Jonsson et al. 2014; Lundin et al. 2011; Pouls et al. 2022). Excluding the outlier, the random effects model indicated a higher relative risk of self‐harm among individuals with ID compared to the general population. Relative risk = 3.16, (95% CI 2.3 to 4.35, *n* = 141 332, *k* = 5, *I* ^2^ = 89%, *τ* ^2^ = 0.12, *p* < 0.001). See Figure 3 for a forest plot illustrating the pooled estimate excluding the outlier.

### 3.5 Sensitivity Analyses

Most of the planned subgroup and sensitivity analyses, as well as all moderator analyses, could not be carried due to the limited number of included studies, as the recommended minimum is 10 (Mathias Harrer et al. 2021). Visual inspection of the funnel plot and Egger's regression test of asymmetry did not indicate possibility of publication bias in the meta‐analyses of suicide (intercept: 1.43, 95% CI −5.96 to 8.81, *t* = 0.38, *p* = 0.724), or self‐harm (intercept: −5.87, 95% CI −16.68 to 4.94, *t* = −1.07, *p* = 0.347), see Figures S1 and S2. However, due to the low number of studies included, the formal analysis of publication bias was underpowered. It was not possible to evaluate Orwin's Fail‐safe N and trim and fill procedures due to too few studies.

## 4 Discussion

While individuals with ID are exposed to many known risk factors for suicide and self‐harm, suicidality in this group is rarely studied. The aim of this systematic review and meta‐analysis was to synthesise research on suicide and self‐harm, including suicide attempts, among individuals with ID compared to the general population. Separate meta‐analyses were conducted with suicide death and self‐harm as the outcomes, including five studies in each analysis. The results indicate that the relative risk of suicide death among people with ID is approximately half that of the general population, while the risk of self‐harm is about three times higher. These findings highlight a paradox: Despite an increased prevalence of self‐harm, individuals with ID appear to have a lower suicide mortality rate than the general population. However, the results should be interpreted with caution due to few primary studies and high heterogeneity between studies.

Although self‐harm is closely linked to suicide in the general population (Runeson et al. 2016), the finding that individuals with ID have a lower suicide risk align with the conclusion of a prior systematic review on ID and suicidality (Dodd et al. 2016). Cognitive impairments in ID may hinder the ability to plan and act on suicidal intent or result in impulsive attempts with lower lethality, while protective factors such as supervised living and limited access to firearms further lower the suicide mortality rate (Cai et al. 2022; Chan and Bhandarkar 2025). Self‐harm may occur for multiple reasons in individuals with ID, with relief from overwhelming emotions, trauma and loss, and difficulty in articulating emotions being the most commonly self‐reported reasons in this population (Samways et al. 2024). Further, stereotypical self‐harm behaviours (e.g., head‐banging, biting, skin‐picking and ingesting non‐food substances) are common in ID, particularly in genetic syndromes linked to ID (Arron et al. 2011; Deb 1998). These self‐harm behaviours often stem from sensory or communicative difficulties, positive social reinforcement, or relief from pain or discomfort (Dimian and Symons 2022; National Institute for Health and Care Excellence 2015; Summers et al. 2017). Thus, self‐harming behaviour likely serves multiple functions for individuals with ID whereas cognitive impairments lower the risk of lethal suicide attempts, which may help explain the lower suicide risk despite higher rates of self‐harm. Although their lower suicide risk, the high prevalence of self‐harm suggests significant psychological distress and unmet mental health needs among individuals with ID, and interventions addressing self‐harm in this population are warranted.

### 4.1 Strengths and Limitations

This is, to the best of our knowledge, the first systematic review with a meta‐analysis investigating the relative risk for suicide death and self‐harm including suicide attempts in individuals with ID. This represents a significant step towards understanding suicidality in this population. The systematic review was done in line with PRISMA guidelines and was pre‐registered on PROSPERO, ensuring transparency.

Nonetheless, some limitations should be mentioned. First, both meta‐analyses had high between‐study heterogeneity and combined studies with different levels of adjustment into one estimate, and it was not possible to explore the sources of heterogeneity in subgroup analyses due to the small number of studies. Second, due to the small number of included studies, it is possible that the outliers identified may reflect clinically relevant differences in the heterogeneous population with ID rather than artefacts from the statistical models or study designs. Third, several post‐preregistration adjustments were necessary (see Supporting Information), introducing assumptions that may have influenced study weighting and the final pooled estimate, though the extent and direction of this impact remain uncertain. Fourth, all included studies were from high‐income countries, and findings may not generalise to low‐ and middle‐income settings where 75% of suicides occur (WHO 2014). Fifth, although both exposure to risk factors for suicide and self‐harm (Fazel and Runeson 2020) and prevalence of these outcomes vary by age (Davis Weaver et al. 2025), it was not possible to study potential age differences in this review. The adjustment for age was inconsistently applied across studies, and the age of participants was often not reported, thus preventing a meta‐analytic evaluation. Sixth, suicidality in the ID population remains complex, and by including self‐harm as a suicidality outcome to capture potential suicidal behaviours, the estimate may be influenced by behaviours without suicidal intent.

### 4.2 Future Directions

Our findings highlight two key challenges in studying suicidality among individuals with ID. First, the heterogeneity the ID population likely influences suicidal tendencies, which was indicated in both meta‐analyses. Those with mild ID, similar to the general population, often live independently and might have a better ability to express suicidal thoughts than individuals with more severe forms of ID (Boat et al. 2015). In contrast, individuals with severe or profound ID may have limited ability to conceptualise or vocalise such thoughts and typically reside in supervised settings with fewer opportunities for self‐harm or suicide (McEvoy et al. 2012). A study including individuals with borderline intellectual functioning—cognitive abilities that fall between the typical range and ID ‐ (Lundin et al. 2011) was an outlier with a higher risk estimate in the meta‐analysis of suicide. Conversely, in the self‐harm meta‐analysis, a study focused on individuals with Down syndrome (Singhal et al. 2014) was an outlier with a lower risk estimate, possibly due to the more structured living conditions in this group. These two studies were identified as outliers and removed from further analyses, but it is possible that they represent clinically relevant sub‐groups or point to factors that affect the risk of suicide and self‐harm in ID, such as ID severity and genetic aetiology. Only one study, however, reported participant distribution by severity (Strauss et al. 1998) and no studies performed sub‐group analyses to evaluate its impact on suicide or self‐harm. Second, it is possible that the relative risk of suicide and self‐harm is underestimated due to diagnostic overshadowing. When determining the cause of death, diagnostic overshadowing could lead to suicides and suicide attempts being recorded as accidents, especially when there was limited prior communication of suicidal intent or when the method used makes intent difficult to determine (e.g., falls from heights and pedestrian collisions) (Chan and Bhandarkar 2025; Landes and Peek 2013). This challenge may be more pronounced in individuals with moderate, severe or profound ID due to difficulties in expressing suicidal thoughts (discussed above); however, this issue needs to be clarified in future studies. All studies except one (Flygare Wallén et al. 2023) were restricted to intentional self‐harm. For instance, two studies found higher mortality from drowning, falls, and other accidents in individuals with ID, where intent is difficult to determine retrospectively (Patja et al. 2001; Strauss et al. 1998). Future studies can address these remaining questions by providing more details on the level of ID and the prevalence of genetic syndromes among study participants, as well as including self‐harm with both determined (i.e., ICD codes X60–X84) and undetermined (i.e., ICD codes Y10–Y34) intent in joint and separate analyses.

## 5 Conclusion

Although it remains a considerably understudied research question, the synthesised literature indicates that the relative risk of suicide among individuals with ID is lower and self‐harm including suicide attempt is higher compared to the general population. These results arise from studies that, with few exceptions, did not distinguish between individuals with different levels of ID severity or different ID presentations, and this heterogeneity points to potential risk differences in specific sub‐groups. Furthermore, individuals with ID are disproportionately exposed to numerous risk factors for suicidality, meaning the individual risk of suicide and self‐harm may vary significantly depending on these factors. Given the gaps in our current understanding, further primary research is needed to better understand the relative risk of suicide in individuals with ID and its causes, by extension informing assessment methods as well as targeted interventions and support strategies.

## Conflicts of Interest

The authors declare no conflicts of interest.

## Review Protocol

The review protocol can be accessed at PROSPERO (https://www.crd.york.ac.uk/PROSPERO/view/CRD42023494017).

## Supporting information


**Data S1:** Supporting Information.

**Data S2:** Supporting Information.

**Data S3:** Supporting Information.

## Data Availability

The data and code used for analyses are uploaded to the Open Science Framework (https://osf.io/xskbc/).
