# Supplementary material for: Suicide and Self‐Harm in Intellectual Disability: A Systematic Review and Meta‐Analysis
Source: J Intellect Disabil Res. 2025 Oct 4;70(4):364–74. doi: 10.1111/jir.70052 (PMC12950635; doi:10.1111/jir.70052)
Supplement: Supplementary file 3 — Data S3: Supporting Information. [file JIR-70-364-s002.docx]

**Supplementary material**

Title: Suicidality in intellectual disability: A systematic review and meta-analysis

**Supplementary material 1. Methodological changes**

From pre-registration (OSF, 2023; PROSPERO, 2023)

**Changes and clarifications in inclusion criteria**

| **Inclusion criteria at pre-reg** | **Change/clarification** |
| --- | --- |
| Observational studies |  |
| Quantitative design |  |
| Case-control data on the relative risk of completed suicide, suicide attempts and self-harm expressed as an odds-, risk- or hazard ratio | Clarification   - specify that the data must include ID as exposure, linked with the outcome   Change   - Relative risk estimate |
| Articles that define the exposure by a diagnosis of ID or MR according to ICD or DSM | Change   - as we noticed that the amount of article was scarce, we broadened our inclusion to also including less robust/less clear presented ways of defining ID/MR |

**New inclusion criteria**

| **Inclusion criteria** | **Exclusion criteria** |
| --- | --- |
| Observational studies | Intervention studies |
| Quantitative design | Psychological autopsy studies |
| A relative risk estimate of completed suicide (outcome). |  |
| Articles that define the exposure by a diagnosis of ID or MR according to ICD or DSM or any other method. |  |
| The data on exposure (ID) and the outcome (suicide) must be linked together. |  |

**Techniques for derives estimates in cases when the data available was not sufficient to perform a meta-analysis**

- - In the cases where person years (not number of participants with ID) were reported, number of person years was divided with the follow-up time. This involves the assumption that no one dies during the follow-up time, resulting in an under-estimation of the number of participants included.
    - This technique was used on the study by Strauss et al. (1998) and Zilber et al. (1989).
  - When only the number of the total population, but not the population with ID was reported, a prevalence of 1 % was assumed. The exact prevalence of ID in the total population is uncertain, with some studies suggesting rates as high as 2–3%. However, the most widely accepted estimate is around 1% (Maulik et al., 2011).
    - This technique was used on the study by Park et al. (2017).
  - For one study, odds ratio was calculated by dividing the odds of an event in the exposed group by the odds of the event in the non-exposed group.
    - This technique was used on the study by Cervantes et al. (2023)
  - When risk estimates were presented for males and females separately, the risk estimates were combined using the method from van Dooren et al. (2013).
    - This technique was used on the study by Patja et al. (2001).

**Supplementary material 2. Documentation of search strategies**

Karolinska Institutet University Library search consultation group

Date: May 24, 2024

Topic/research question: Completed suicide, suicide attempts and self-injurious behaviour among people with intellectual disability.

Name of researcher(s): Oskar Flygare, Department of Clinical Neuroscience, KI

Librarian(s): Ingrid Andersson and Sabina Gillsund, KI University Library

Databases:

1. Medline (Ovid)
2. Embase (Elsevier)
3. Web of Science (Clarivate)
4. PsycInfo (EBSCO)

Total number of hits:

- Before deduplication: 11,523
- After deduplication: 6,793

1. Medline

| Interface: Ovid MEDLINE(R) ALL  Date of Search: May 24, 2024  Number of hits: 2,955  Comment: In Ovid, two or more words are automatically searched as phrases; i.e. no quotation marks are needed | Field labels   - exp/ = exploded MeSH term - / = non exploded MeSH term - .ti,ab,kf. = title, abstract and author keywords - adjx = within x words, regardless of order - * = truncation of word for alternate endings |
| --- | --- |
| Database(s): **Ovid MEDLINE(R) ALL**1946 to May 23, 2024 Search Strategy:   \| **No.** \| **Searches** \| **Results** \| \| --- \| --- \| --- \| \| 1 \| exp Self-Injurious Behavior/ \| 86 434 \| \| 2 \| exp Intellectual Disability/mo \| 619 \| \| 3 \| (suicid* or automutilat* or nonsuicid* or parasuicid* or selfburn* or selfcut* or selfdestruct* or self-directed violen* or selfharm* or selfimmolat* or selfincinerat* or selfinflict* or selfinjur* or selfmutilat* or selfpoison* or selfwound* or fatal attempt*).ti,ab,kf. \| 103 193 \| \| 4 \| ((self or selv* or themsel* or herself or himself or oneself) adj3 (harm* or injur* or destruct* or mutilat* or kill* or death* or die or dying or dead or burn* or cut* or immolat* or incinerat* or inflict* or poison* or wound*)).ti,ab,kf. \| 31 969 \| \| 5 \| 1 or 2 or 3 or 4 \| 143 166 \| \| 6 \| exp Intellectual Disability/ \| 107 451 \| \| 7 \| (intellect* adj3 (disorder* or deficienc* or deficit* or disabilit* or dysfunction* or impair* or incapacit* or retard* or handicap*)).ti,ab,kf. \| 33 069 \| \| 8 \| (cognitive retard* or idiocy or mental* deficienc* or mental* deficit* or mental* disabilit* or mental* handicap* or mental* impair* or mental* incapacit* or mental* retard*).ti,ab,kf. \| 45 662 \| \| 9 \| (adrenoleukodystroph* or lissencephal* or subcortical band heterotop* or Cri-du-Chat* or Cat Cry Syndrome* or Crying Cat Syndrome* or Typus Degenerativus Amstelodamen* or X-Linked CdLS* or Mongolism* or glycogen storage disease* or mucopolysaccharidosis ii or pyruvate dehydrogenase complex deficienc * or "47,XY,+21" or Trisomy or "47,XX,+21" or Chromosome 13 Duplication* or "Chromosome 11p13 Deletion*" or "Chromosome 7q11.23 Deletion*" or Alpers or Danon or "De Sanctis" or "GM3 synthase deficienc*" or "guanidinoacetate methyltransferase" or Hennekam* or hypermethioninemia* or "Leigh disease*" or leprechauni* or mannosidos* or "maple syrup urine" or "ornithine transcarbamylase*" or "osteopetrosis with renal tubular acido*" or "prolidase deficienc*" or "Sandhoff disease*" or "sialidosis type 2").ti,ab,kf. \| 35 683 \| \| 10 \| ((5p or De Lange* or down* or Willi* or royer* or Rubinstein* or Broad Thumb* or Patau* or WAGR* or Wilms Tumor* or 11p Partial Monosomy or Williams* or Beuren* or Aortic Stenos* or fragile x or lesch-nyhan or coffin-lowry or menkes kinky hair or rett or Dubowitz or "Ellis van Creveld" or Hunter or Hurler or Kabuki makeup or Marinesco Sjogren or Maroteaux Lamy or Peters plus* or Sanjad-Sakati* or Schinzel-Giedion* or Shprintzen-Goldberg* or Weismann Netter*) adj3 (syndrome* or complex*)).ti,ab,kf. \| 51 539 \| \| 11 \| 6 or 7 or 8 or 9 or 10 \| 180 749 \| \| 12 \| 5 and 11 \| 3 005 \| \| **13** \| **12 NOT (exp animals/ not humans.sh.)** \| **2 955** \| | |

2. Embase

| Interface: embase.com  Date of Search: May 24, 2024  Number of hits: 3,531  Comment: Emtree is the controlled vocabulary in Embase | Field labels   - /exp = exploded Emtree term - /de = non exploded Emtree term - ti,ab,kw = title, abstract and author keywords - NEAR/x = within x words, regardless of order - * = truncation of word for alternate endings |
| --- | --- |
| \| **No.** \| **Searches** \| **Results** \| \| --- \| --- \| --- \| \| #01 \| 'suicidal behavior'/exp \| 138 408 \| \| #02 \| 'automutilation'/exp \| 27 114 \| \| #03 \| suicid*:ti,ab,kw OR automutilat*:ti,ab,kw OR nonsuicid*:ti,ab,kw OR parasuicid*:ti,ab,kw OR selfburn*:ti,ab,kw OR selfcut*:ti,ab,kw OR selfdestruct*:ti,ab,kw OR 'self-directed violen*':ti,ab,kw OR selfharm*:ti,ab,kw OR selfimmolat*:ti,ab,kw OR selfincinerat*:ti,ab,kw OR selfinflict*:ti,ab,kw OR selfinjur*:ti,ab,kw OR selfmutilat*:ti,ab,kw OR selfpoison*:ti,ab,kw OR selfwound*:ti,ab,kw OR 'fatal attempt*':ti,ab,kw \| 149 983 \| \| #04 \| ((self OR selv* OR themsel* OR herself OR himself OR oneself) NEAR/3 (harm* OR injur* OR destruct* OR mutilat* OR kill* OR death* OR die OR dying OR dead OR burn* OR cut* OR immolat* OR incinerat* OR inflict* OR poison* OR wound*)):ti,ab,kw \| 40 929 \| \| #05 \| #1 OR #2 OR #3 OR #4 \| 201 394 \| \| #06 \| 'mental deficiency'/exp \| 170 193 \| \| #07 \| (intellect* NEAR/3 (disorder* OR deficienc* OR deficit* OR disabilit* OR dysfunction* OR impair* OR incapacit* OR retard* OR handicap*)):ti,ab,kw \| 47 266 \| \| #08 \| 'cognitive retard*':ti,ab,kw OR idiocy:ti,ab,kw OR 'mental* deficienc*':ti,ab,kw OR 'mental* deficit*':ti,ab,kw OR 'mental* disabilit*':ti,ab,kw OR 'mental* handicap*':ti,ab,kw OR 'mental* impair*':ti,ab,kw OR 'mental* incapacit*':ti,ab,kw OR 'mental* retard*':ti,ab,kw \| 57 344 \| \| #09 \| adrenoleukodystroph*:ti,ab,kw OR lissencephal*:ti,ab,kw OR 'subcortical band heterotop*':ti,ab,kw OR 'cri du chat*':ti,ab,kw OR 'cat cry syndrome*':ti,ab,kw OR 'crying cat syndrome*':ti,ab,kw OR 'typus degenerativus amstelodamen*':ti,ab,kw OR 'x-linked cdls*':ti,ab,kw OR mongolism*:ti,ab,kw OR 'glycogen storage disease*':ti,ab,kw OR 'mucopolysaccharidosis ii':ti,ab,kw OR 'pyruvate dehydrogenase complex deficienc*':ti,ab,kw OR 47,xy,+21:ti,ab,kw OR trisomy:ti,ab,kw OR 47,xx,+21:ti,ab,kw OR 'chromosome 13 duplication*':ti,ab,kw OR 'chromosome 11p13 deletion*':ti,ab,kw OR 'chromosome 7q11.23 deletion*':ti,ab,kw OR alpers:ti,ab,kw OR danon:ti,ab,kw OR 'de sanctis':ti,ab,kw OR 'gm3 synthase deficienc*':ti,ab,kw OR 'guanidinoacetate methyltransferase':ti,ab,kw OR hennekam*:ti,ab,kw OR hypermethioninemia*:ti,ab,kw OR 'leigh disease*':ti,ab,kw OR leprechauni*:ti,ab,kw OR mannosidos*:ti,ab,kw OR 'maple syrup urine':ti,ab,kw OR 'ornithine transcarbamylase*':ti,ab,kw OR 'osteopetrosis with renal tubular acido*':ti,ab,kw OR 'prolidase deficienc*':ti,ab,kw OR 'sandhoff disease*':ti,ab,kw OR 'sialidosis type 2':ti,ab,kw \| 49 877 \| \| #10 \| ((5p OR 'de lange*' OR down* OR willi* OR royer* OR rubinstein* OR 'broad thumb*' OR patau* OR wagr* OR 'wilms tumor*' OR '11p partial monosomy' OR williams* OR beuren* OR 'aortic stenos*' OR 'fragile x' OR 'lesch nyhan' OR 'coffin lowry' OR 'menkes kinky hair' OR rett OR dubowitz OR 'ellis van creveld' OR hunter OR hurler OR 'kabuki makeup' OR 'marinesco sjogren' OR 'maroteaux lamy' OR 'peters plus*' OR 'sanjad sakati*' OR 'schinzel giedion*' OR 'shprintzen goldberg*' OR 'weismann netter*') NEAR/3 (syndrome* OR complex*)):ti,ab,kw \| 68 625 \| \| #11 \| #6 OR #7 OR #8 OR #9 OR #10 \| 272 892 \| \| #12 \| #5 AND #11 \| 4 156 \| \| #13 \| #12 NOT ([animals]/lim NOT [humans]/lim) \| 4 074 \| \| **#14** \| **#13 NOT ('chapter'/it OR 'conference abstract'/it OR 'conference review'/it)** \| **3 531** \| | |

3. Web of Science Core Collection

| Interface: Clarivate Analytics  Editions = A&HCI , ESCI , SCI-EXPANDED , SSCI  Date of Search: May 24, 2024  Number of hits: 2,341 | Field labels   - TS/Topic = title, abstract, author keywords and Keywords Plus - NEAR/x = within x words, regardless of order - * = truncation of word for alternate endings   Note: the *Exact search*-function was used for all the searches |
| --- | --- |
| \| **No.** \| **Searches** \| **Results** \| \| --- \| --- \| --- \| \| 1 \| TS=((suicid* OR automutilat* OR nonsuicid* OR parasuicid* OR selfburn* OR selfcut* OR selfdestruct* OR "self-directed violen*" OR selfharm* OR selfimmolat* OR selfincinerat* OR selfinflict* OR selfinjur* OR selfmutilat* OR selfpoison* OR selfwound* OR "fatal attempt*")) \| 131 753 \| \| 2 \| TS=(((self OR selv* OR themsel* OR herself OR himself OR oneself) NEAR/3 (harm* OR injur* OR destruct* OR mutilat* OR kill* OR death* OR die OR dying OR dead OR burn* OR cut* OR immolat* OR incinerat* OR inflict* OR poison* OR wound*))) \| 47 572 \| \| 3 \| #1 OR #2 \| 163 155 \| \| 4 \| TS=((intellect* NEAR/3 (disorder* OR deficienc* OR deficit* OR disabilit* OR dysfunction* OR impair* OR incapacit* OR retard* OR handicap*))) \| 50 339 \| \| 5 \| TS=(("cognitive retard*" OR idiocy OR "mental* deficienc*" OR "mental* deficit*" OR "mental* disabilit*" OR "mental* handicap*" OR "mental* impair*" OR "mental* incapacit*" OR "mental* retard*")) \| 54 883 \| \| 6 \| TS=((adrenoleukodystroph* OR lissencephal* OR "subcortical band heterotop*" OR Cri-du-Chat* OR "Cat Cry Syndrome*" OR "Crying Cat Syndrome*" OR "Typus Degenerativus Amstelodamen*" OR "X-Linked CdLS*" OR Mongolism* OR "glycogen storage disease*" OR "mucopolysaccharidosis ii" OR "pyruvate dehydrogenase complex deficienc *" OR 47,XY,+21 OR Trisomy OR 47,XX,+21 OR "Chromosome 13 Duplication*" OR "Chromosome 11p13 Deletion*" OR "Chromosome 7q11.23 Deletion*" OR Alpers OR Danon OR "De Sanctis" OR "GM3 synthase deficienc*" OR "guanidinoacetate methyltransferase" OR Hennekam* OR hypermethioninemia* OR "Leigh disease*" OR leprechauni* OR mannosidos* OR "maple syrup urine" OR "ornithine transcarbamylase*" OR "osteopetrosis with renal tubular acido*" OR "prolidase deficienc*" OR "Sandhoff disease*" OR "sialidosis type 2")) \| 43 252 \| \| 7 \| TS=(((5p OR "De Lange*" OR down* OR Willi* OR royer* OR Rubinstein* OR "Broad Thumb*" OR Patau* OR WAGR* OR "Wilms Tumor*" OR "11p Partial Monosomy" OR Williams* OR Beuren* OR "Aortic Stenos*" OR "fragile x" OR lesch-nyhan OR coffin-lowry OR "menkes kinky hair" OR rett OR Dubowitz OR "Ellis van Creveld" OR Hunter OR Hurler OR "Kabuki makeup" OR "Marinesco Sjogren" OR "Maroteaux Lamy" OR "Peters plus*" OR Sanjad-Sakati* OR Schinzel-Giedion* OR Shprintzen-Goldberg* OR "Weismann Netter*") NEAR/3 (syndrome* OR complex*))) \| 74 366 \| \| 8 \| #4 OR #5 OR #6 OR #7 \| 194 608 \| \| **9** \| **#3 AND #8** \| **2 341** \| | |

4. Psycinfo

| Interface: EBSCO  Date of Search: May 24, 2024  Number of hits: 2,696 | Field labels   - DE = subject heading - TI = title - AB = abstract - KW = author keywords - Nx = within x words, regardless of order - * = truncation of word for alternate endings   Note: sometimes “quotation marks” are needed for single search terms to avoid automatic term mapping (lemmatization) |
| --- | --- |
| \| **No.** \| **Searches** \| **Results** \| \| --- \| --- \| --- \| \| S01 \| (DE "Self-Destructive Behavior" OR DE "Nonsuicidal Self-Injury" OR DE "Suicidal Behavior" OR DE "Head Banging" OR DE "Self-Inflicted Wounds" OR DE "Self-Poisoning" OR DE "Attempted Suicide" OR DE "Suicidal Ideation" OR DE "Suicide" OR DE "Military Suicide" OR DE "Youth Suicide" OR DE "Suicidality" OR DE "Suicide Prevention" OR DE "Suicidology") \| 62 893 \| \| S02 \| TI ((suicid* or automutilat* or nonsuicid* or parasuicid* or selfburn* or selfcut* or selfdestruct* or self-directed violen* or selfharm* or selfimmolat* or selfincinerat* or selfinflict* or selfinjur* or selfmutilat* or selfpoison* or selfwound* or "fatal attempt*")) OR AB ((suicid* or automutilat* or nonsuicid* or parasuicid* or selfburn* or selfcut* or selfdestruct* or self-directed violen* or selfharm* or selfimmolat* or selfincinerat* or selfinflict* or selfinjur* or selfmutilat* or selfpoison* or selfwound* or "fatal attempt*")) OR KW ((suicid* or automutilat* or nonsuicid* or parasuicid* or selfburn* or selfcut* or selfdestruct* or self-directed violen* or selfharm* or selfimmolat* or selfincinerat* or selfinflict* or selfinjur* or selfmutilat* or selfpoison* or selfwound* or "fatal attempt*")) \| 81 822 \| \| S03 \| TI ( ((self or selv* or themsel* or herself or himself or oneself) N3 (harm* or injur* or destruct* or mutilat* or kill* or death* or die or dying or dead or burn* or cut* or immolat* or incinerat* or inflict* or poison* or wound*)) ) OR AB ( ((self or selv* or themsel* or herself or himself or oneself) N3 (harm* or injur* or destruct* or mutilat* or kill* or death* or die or dying or dead or burn* or cut* or immolat* or incinerat* or inflict* or poison* or wound*)) ) OR KW ( ((self or selv* or themsel* or herself or himself or oneself) N3 (harm* or injur* or destruct* or mutilat* or kill* or death* or die or dying or dead or burn* or cut* or immolat* or incinerat* or inflict* or poison* or wound*)) ) \| 28 336 \| \| S04 \| S1 OR S2 OR S3 \| 100 771 \| \| S05 \| (DE "Intellectual Development Disorder" OR DE "Anencephaly" OR DE "Crying Cat Syndrome" OR DE "Down's Syndrome" OR DE "Tay Sachs Disease" OR DE "Developmental Disabilities" OR DE "Specific Language Impairment" OR DE "Cognitive Impairment" OR DE "Sex Linked Hereditary Disorders" OR DE "Androgen Insensitivity Syndrome" OR DE "Fragile X Syndrome" OR DE "Hemophilia" OR DE "Turners Syndrome" OR DE "Fragile X Syndrome" OR DE "Prader Willi Syndrome" OR DE "Rett Syndrome" OR DE "Williams Syndrome" OR DE "Cornelia De Lange Syndrome") \| 118 130 \| \| S06 \| TI ( (intellect* N3 (disorder* or deficienc* or deficit* or disabilit* or dysfunction* or impair* or incapacit* or retard* or handicap*)) ) OR AB ( (intellect* N3 (disorder* or deficienc* or deficit* or disabilit* or dysfunction* or impair* or incapacit* or retard* or handicap*)) ) OR KW ( (intellect* N3 (disorder* or deficienc* or deficit* or disabilit* or dysfunction* or impair* or incapacit* or retard* or handicap*)) ) \| 26 938 \| \| S07 \| TI (("cognitive retard*" OR idiocy OR "mental* deficienc*" OR "mental* deficit*" OR "mental* disabilit*" OR "mental* handicap*" OR "mental* impair*" OR "mental* incapacit*" OR "mental* retard*")) OR AB (("cognitive retard*" OR idiocy OR "mental* deficienc*" OR "mental* deficit*" OR "mental* disabilit*" OR "mental* handicap*" OR "mental* impair*" OR "mental* incapacit*" OR "mental* retard*")) OR KW (("cognitive retard*" OR idiocy OR "mental* deficienc*" OR "mental* deficit*" OR "mental* disabilit*" OR "mental* handicap*" OR "mental* impair*" OR "mental* incapacit*" OR "mental* retard*")) \| 48 149 \| \| S08 \| TI ((adrenoleukodystroph* or lissencephal* or "subcortical band heterotop*" or "Cri-du-Chat*" or "Cat Cry Syndrome*" or "Crying Cat Syndrome*" or "Typus Degenerativus Amstelodamen*" or "X-Linked CdLS*" or Mongolism* or "glycogen storage disease" or "mucopolysaccharidosis ii" or "pyruvate dehydrogenase complex deficienc*" or "47,XY,+21" or "Trisomy" or "47,XX,+21" or "Chromosome 13 Duplication*" or "Chromosome 11p13 Deletion*" or "Chromosome 7q11.23 Deletion*" OR Alpers OR Danon OR "De Sanctis" OR "GM3 synthase deficienc*" OR "guanidinoacetate methyltransferase" OR Hennekam* OR hypermethioninemia* OR "Leigh disease*" OR leprechauni* OR mannosidos* OR "maple syrup urine" OR "ornithine transcarbamylase*" OR "osteopetrosis with renal tubular acido*" OR "prolidase deficienc*" OR "Sandhoff disease*" OR "sialidosis type 2")) OR AB ((adrenoleukodystroph* or lissencephal* or "subcortical band heterotop*" or "Cri-du-Chat*" or "Cat Cry Syndrome*" or "Crying Cat Syndrome*" or "Typus Degenerativus Amstelodamen*" or "X-Linked CdLS*" or Mongolism* or "glycogen storage disease" or "mucopolysaccharidosis ii" or "pyruvate dehydrogenase complex deficienc*" or "47,XY,+21" or "Trisomy" or "47,XX,+21" or "Chromosome 13 Duplication*" or "Chromosome 11p13 Deletion*" or "Chromosome 7q11.23 Deletion*" OR Alpers OR Danon OR "De Sanctis" OR "GM3 synthase deficienc*" OR "guanidinoacetate methyltransferase" OR Hennekam* OR hypermethioninemia* OR "Leigh disease*" OR leprechauni* OR mannosidos* OR "maple syrup urine" OR "ornithine transcarbamylase*" OR "osteopetrosis with renal tubular acido*" OR "prolidase deficienc*" OR "Sandhoff disease*" OR "sialidosis type 2")) OR KW ((adrenoleukodystroph* or lissencephal* or "subcortical band heterotop*" or "Cri-du-Chat*" or "Cat Cry Syndrome*" or "Crying Cat Syndrome*" or "Typus Degenerativus Amstelodamen*" or "X-Linked CdLS*" or Mongolism* or "glycogen storage disease" or "mucopolysaccharidosis ii" or "pyruvate dehydrogenase complex deficienc*" or "47,XY,+21" or "Trisomy" or "47,XX,+21" or "Chromosome 13 Duplication*" or "Chromosome 11p13 Deletion*" or "Chromosome 7q11.23 Deletion*" OR Alpers OR Danon OR "De Sanctis" OR "GM3 synthase deficienc*" OR "guanidinoacetate methyltransferase" OR Hennekam* OR hypermethioninemia* OR "Leigh disease*" OR leprechauni* OR mannosidos* OR "maple syrup urine" OR "ornithine transcarbamylase*" OR "osteopetrosis with renal tubular acido*" OR "prolidase deficienc*" OR "Sandhoff disease*" OR "sialidosis type 2")) \| 2 311 \| \| S09 \| TI (((5p OR "De Lange*" OR down* OR Willi* OR royer* OR Rubinstein* OR "Broad Thumb*" OR Patau* OR WAGR* OR "Wilms Tumor*" OR "11p Partial Monosomy" OR Williams* OR Beuren* OR "Aortic Stenos*" OR "fragile x" OR lesch-nyhan OR coffin-lowry OR "menkes kinky hair" OR rett OR Dubowitz OR "Ellis van Creveld" OR Hunter OR Hurler OR "Kabuki makeup" OR "Marinesco Sjogren" OR "Maroteaux Lamy" OR "Peters plus*" OR Sanjad-Sakati* OR Schinzel-Giedion* OR Shprintzen-Goldberg* OR "Weismann Netter*") N3 (syndrome* OR complex*))) OR AB (((5p OR "De Lange*" OR down* OR Willi* OR royer* OR Rubinstein* OR "Broad Thumb*" OR Patau* OR WAGR* OR "Wilms Tumor*" OR "11p Partial Monosomy" OR Williams* OR Beuren* OR "Aortic Stenos*" OR "fragile x" OR lesch-nyhan OR coffin-lowry OR "menkes kinky hair" OR rett OR Dubowitz OR "Ellis van Creveld" OR Hunter OR Hurler OR "Kabuki makeup" OR "Marinesco Sjogren" OR "Maroteaux Lamy" OR "Peters plus*" OR Sanjad-Sakati* OR Schinzel-Giedion* OR Shprintzen-Goldberg* OR "Weismann Netter*") N3 (syndrome* OR complex*))) OR KW (((5p OR "De Lange*" OR down* OR Willi* OR royer* OR Rubinstein* OR "Broad Thumb*" OR Patau* OR WAGR* OR "Wilms Tumor*" OR "11p Partial Monosomy" OR Williams* OR Beuren* OR "Aortic Stenos*" OR "fragile x" OR lesch-nyhan OR coffin-lowry OR "menkes kinky hair" OR rett OR Dubowitz OR "Ellis van Creveld" OR Hunter OR Hurler OR "Kabuki makeup" OR "Marinesco Sjogren" OR "Maroteaux Lamy" OR "Peters plus*" OR Sanjad-Sakati* OR Schinzel-Giedion* OR Shprintzen-Goldberg* OR "Weismann Netter*") N3 (syndrome* OR complex*))) \| 14 784 \| \| S10 \| S5 OR S6 OR S7 OR S8 OR S9 \| 141 111 \| \| **S11** \| **S4 AND S10** \| **2 696** \| | |

**Supplementary material 2. Risk of Bias assessment.**

| **Study** | **Bias from confounding** | **Bias from exposure measure** | **Bias from dropout** | **Bias from outcome measure** | **Bias from selective reporting** | **Conflict of interest** | **Overall assessment** | **Comment** |
| --- | --- | --- | --- | --- | --- | --- | --- | --- |
| Cervantes et al., 2023 | High | Low | Low | Low | Low | No | Low | The lack of adjustment for important confounders is not expected to lead to bias. |
| Erlangsen et al., 2020 | Low | Low | Low | Low | Low | No | Low |  |
| Flygare Wallen et al., 2023 | High | Low | Low | Low | Low | No | Low | The lack of adjustment for important confounders is not expected to lead to bias. |
| Jonsson et al., 2014 | Low | Low | Low | Low | Low | No | Low |  |
| Park et al., 2017 | Low | Low | Low | Low | Low | No | Low |  |
| Patja et al., 2001 | High | Low | Low | Low | Low | Unclear | Low | The lack of adjustment for important confounders is not expected to lead to bias. |
| Pouls et al., 2022 | High | High | Low | High | Low | No | High |  |
| Singhal et al., 2014 | Low | High | Low | High | High | No | High |  |
| Strauss et al., 1998 | High | Low | Unclear | Low | Low | Unclear | Low | The lack of info on non-response is not considered to contribute to significant bias. |
| Lundin et al., 2011 | High | High | High | Low | Low | No | High |  |
| Zilber et al., 1989 | High | Low | Unclear | Low | Low | Unclear | Low | The lack of adjustment for important confounders is not expected to lead to bias. |

Higgins JPT, Morgan RL, Rooney AA, Taylor KW, Thayer KA, Raquel A. Silva RA, Courtney Lemeris C, Akl EA, Bateson TF, Berkman ND, Glenn BS, Hróbjartsson A, LaKind JS, McAleenan A, Meerpohl JJ, Nachman RM, Obbagy JE, O'Connor A, Radke EG, Savović J, Schünemann HJ, Shea B, Tilling K, Verbeek J, Viswanathan M, Sterne JAC. A tool to assess risk of bias in non-randomized follow-up studies of exposure effects (ROBINS-E). *Environment International* 2024 (published online Mar 24); doi: [10.1016/j.envint.2024.108602](https://www.sciencedirect.com/science/article/pii/S0160412024001880).


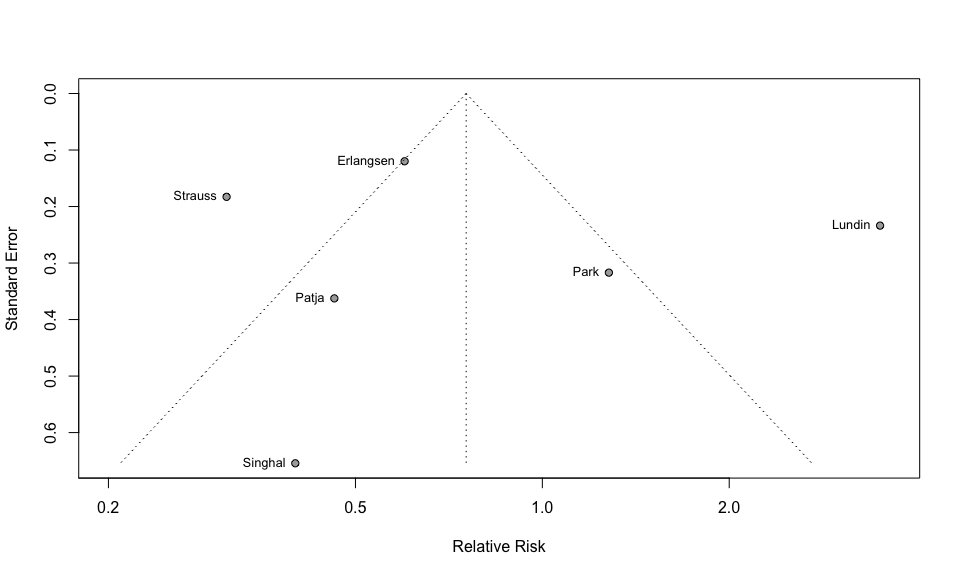


eFigure 1: Funnel plot of included studies, suicide meta-analysis


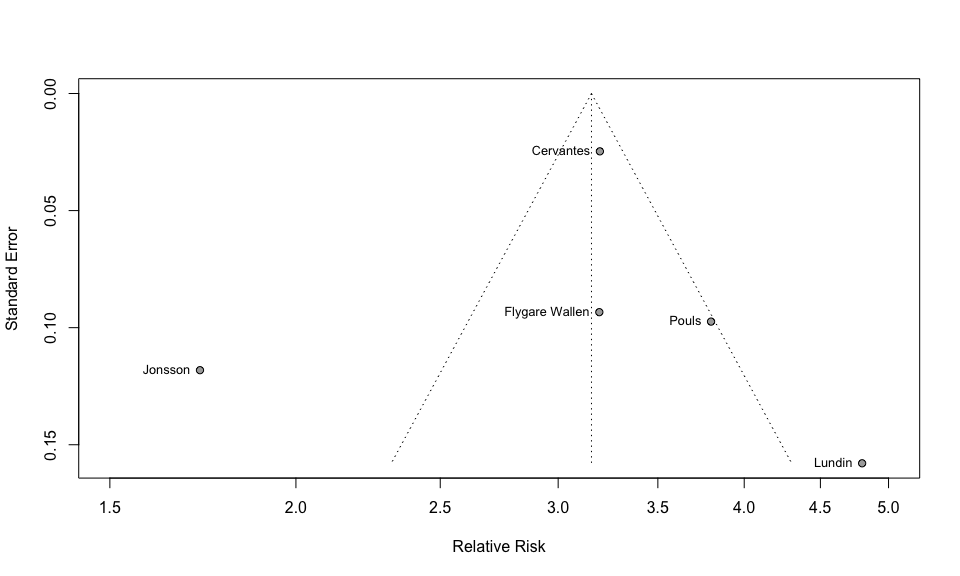


eFigure 2: Funnel plot of included studies, self-harm meta-analysis
